# Early Life Exposure to Tumor Necrosis Factor Induces Precocious Sensorimotor Reflexes Acquisition and Increases Locomotor Activity During Mouse Postnatal Development

**DOI:** 10.3389/fnbeh.2022.845458

**Published:** 2022-03-14

**Authors:** Cristina Paraschivescu, Susana Barbosa, Juliette Van Steenwinckel, Pierre Gressens, Nicolas Glaichenhaus, Laetitia Davidovic

**Affiliations:** ^1^Centre National de la Recherche Scientifique, Institut de Pharmacologie Moléculaire et Cellulaire, Université Côte d’Azur, Valbonne, France; ^2^NeuroDiderot, Inserm, Université de Paris, Paris, France

**Keywords:** TNF, neurodevelopmental disorders, reflex acquisition, behavior, cytokine

## Abstract

Inflammation appears as a cardinal mediator of the deleterious effect of early life stress exposure on neurodevelopment. More generally, immune activation during the perinatal period, and most importantly elevations of pro-inflammatory cytokines levels could contribute to psychopathology and neurological deficits later in life. Cytokines are also required for normal brain function in homeostatic conditions and play a role in neurodevelopmental processes. Despite these latter studies, whether pro-inflammatory cytokines such as Tumor Necrosis Factor (TNF) impact neurodevelopmental trajectories and behavior during the immediate postnatal period remains to be elucidated. To address this issue, we have injected mouse pups daily with recombinant TNF from postnatal day (P)1 to P5. This yielded a robust increase in peripheral and central TNF at P5, and also an increase of additional pro-inflammatory cytokines. Compared to control pups injected with saline, mice injected with TNF acquired the righting and the acoustic startle reflexes more rapidly and exhibited increased locomotor activity 2 weeks after birth. Our results extend previous work restricted to adult behaviors and support the notion that cytokines, and notably TNF, modulate early neurodevelopmental trajectories.

## Introduction

Early life stress (ELS) exposure enhances susceptibility to neurodevelopmental disorders. Inflammation appears as a cardinal mediator of the deleterious effect of ELS on neurodevelopmental trajectories (Cattane et al., 2020). More generally, immune activation during the perinatal period, and most importantly elevations of pro-inflammatory cytokines levels could contribute to psychopathology and neurological deficits later in life (Cattane et al., 2020). Tumor Necrosis Factor (TNF) is a pro-inflammatory cytokine historically known as a chief orchestrator of the innate immune response (Holbrook et al., 2019), *via* signaling through two membrane receptors, TNFR1 and TNFR2. TNF is expressed as a 27 kDa transmembrane form (mTNF) that can be cleaved into a soluble 17 kDa form (sTNF) released in tissues and blood (Kriegler et al., 1988). mTNF signals through both TNFR1 and TNFR2, while sTNF only signals through TNFR1 (Gough and Myles, 2020). TNF and its receptors are also expressed outside the immune compartment, and notably in the brain (Probert, 2015). Cells of the brain parenchyma (neural stem cells, neuronal progenitors, neurons, oligodendrocytes, astrocytes and microglia), as well as endothelial cells of the blood-brain-barrier (BBB) express TNF and its receptors (Probert, 2015).

In animal studies, immune activation induces a surge in both peripheral and central pro-inflammatory cytokines, including TNF (Probert, 2015). This is accompanied by neuroinflammation and behavioral changes collectively known as sickness behavior, which range from anxiodepressive-like behaviors, hypolocomotion, cognitive, and social deficits (Dantzer et al., 2008). In adult rodents, the sole intracerebroventricular injection of TNF recapitulates the hallmarks of sickness behavior (Connor et al., 1998; Kaster et al., 2012), while central blockade of TNF with etanercept alleviated sickness behavior induced by lipopolysaccharides (LPS) (Camara et al., 2015). Also, the acute intraperitoneal injection of TNF induced sickness behavior associated with a spiked increase in the brain levels of TNF, IL-6, and CCL2, as well as astrocytic and microglial activation (Hayley et al., 2002; Biesmans et al., 2015). Finally, TNF was shown to mediate synaptic and learning deficits after systemic poly(I:C) immune challenge, by increasing dendritic spine elimination in the cortex and altering motor learning processes (Garre et al., 2017).

These neuroinflammatory conditions, in which massive induction of TNF in the brain appears deleterious for brain function and behavior, differ from physiological conditions, in which TNF is constitutively secreted in minute amounts by neurons and glia (Probert, 2015). In these conditions, TNF is required for brain cell maintenance and homeostasis (Probert, 2015). Notably, TNF promotes proliferation of oligodendrocyte progenitors and remyelination (Arnett et al., 2001). Also, TNF enhances hippocampal, cortical and striatal synaptic scaling, a form of homeostatic plasticity that enables adjustment of synaptic strength at the neuron-scale in response to sustained activity (Beattie et al., 2002; Santello et al., 2011; Lewitus et al., 2014). Behavioral studies in *Tnf-*knockout (KO) mice have shown that maternal TNF determined the fear response of the adult offspring (Zupan et al., 2017), as well as spatial memory associated with increased hippocampal neurogenesis (Liu et al., 2014).

Tumor necrosis factor also has neuromodulatory function during early neurodevelopment. In mice, TNF is expressed in various brain regions during the first two postnatal weeks of life, a time of active neurogenesis and synaptogenesis (Garay et al., 2013). Moreover, low doses of TNF promote the survival, proliferation, and neuronal differentiation of mouse neonatal neural precursor cells cultures, while higher doses were apoptotic (Bernardino et al., 2008). Furthermore, young *Tnf-*KO mice exhibit an accelerated maturation of the dentate gyrus hippocampal region, but with pyramidal neurons harboring a smaller dendritic arborization in CA1 and CA3 regions (Golan et al., 2004). Finally, both *in vitro* and *in vivo* studies have shown that developing pyramidal neurons from the cortex of *Tnf-*KO mice are deficient in synaptic scaling, that is critical for the activity-dependent refinement of neural circuitry during early development (Stellwagen and Malenka, 2006; Kaneko et al., 2008; Ranson et al., 2012).

This suggests a role for TNF in shaping the nervous system during early developmental stages and that elevated TNF levels triggered by ELS could impact neurodevelopment. However, the consequences of TNF loss-of-function or gain-of-function has been exclusively studied in adult animals and the impact of TNF on behavior in the early postnatal period has not been investigated so far. We hypothetized that TNF gain-of-function during an early postnatal window could impact developmental trajectories and pups behavior. We therefore studied the impact of TNF gain-of-function in the perinatal period on systemic and central cytokine levels and behavior of mouse pups.

## Materials and Methods

### Animals and Treatments

Animal housing and experimentation were conducted in facilities certified by regional authorities (Direction Départementale de Protection des Populations des Alpes-Maritimes, accreditation #C-06-152-5). The study was in accordance to procedures approved by the Ministère de l’Enseignement Supérieur et de la Recherche (APAFIS#19129-201902071212672). Timed-mated OF1 female mice [Charles River (L’Arbresles, France)] were purchased at 15.5 days of gestation [corresponding to embryonic day (E) 15.5 post-conception]. Mice were housed in a temperature (22–24°C) and hygrometry (70–80%)-controlled room with a 12 h light/dark cycle (lights on from 8:00 a.m. to 8:00 p.m.) with *ad libitum* access to water and food (standard chow, reference 4RF25, Mucedola, Milan, Italy). Pregnant dams were housed into individual cages and remained in the same cages until the end of the experiment. The day and time of birth were recorded and only pups born on E19.5 were used. The day of birth was considered as postnatal day 0 (P0). At P1, pups were sexed and only male pups were used for the experiments described here forth. Since both epidemiological and animal studies suggest sex differences in responses to early adversity and a higher susceptibility of males to neurodevelopmental disorders (Davis and Pfaff, 2014), only males were considered in this exploratory study.

Environmental factors, maternal effects, and litter effects are acknowledged confounding variables in developmental and behavioral studies. To overcome the maternal prenatal effect, litters of 12–14 male pups from distinct mothers were randomly formed and fostered to new single mothers. Each litter was housed individually with its newly reassigned mother. Also, the conditions tested (TNF doses or vehicle treatment), were represented in each individual litter. To keep track of the treatment group, pups were labeled on the back according to their group with distinct colors using ink odorless markers. Marking was renewed every other day to guarantee clear identification over the course of the experiments. In doing so, we obtained both TNF-treated and control pups bred by the same mother, thus excluding the confounding effect of differential maternal care or milk quality, that could contribute to developmental or behavioral differences. In the pilot experiment used to determine the optimal TNF dose to be used, pups were injected intraperitoneally (under the iliac fossa, on the right side of the pup, between 10 and 12 a.m) with either carrier- and endotoxin-free recombinant murine soluble TNF (#575204, Biolegend, London, United Kingdom) diluted in sterile PBS, or PBS as vehicle for the control group. We administered 5 μL of solution per pup, with the concentration of 0.25, 1, 5, and 20 μg/Kg adjusted to a standard pup weight for each day of the treatment. Pups were treated once a day from P1 to P5. At P5, body mass (BM) was recorded and pups were sacrificed 3 h post TNF injection by decapitation, then blood and brain were collected.

In further experiments, we used the highest TNF dose of 20 μg/Kg and three independent cohorts were generated and subjected to behavioral testing. Again in this set of experiments, to control for the litter effect, newly formed litters were randomly assigned to new mothers and half of each litter was randomly assigned to the two treatment groups (TNF or vehicle). To keep track of treatment group, each pup was labeled on the back according to its treatment group. Altogether 46 pups were injected with TNF and 47 with PBS, once a day for 5 days, from P1 to P5.

### Pups Behavioral Phenotyping

The acquisition of developmental milestones and reflexes ontology as well as early life behavior were assessed using procedures derived from Fox’s battery of tests and Wahlsten’s adaption of Fox’s tests (Fox, 1965), as described in Heyser (2004) and Feather-Schussler and Ferguson (2016). Pups were tested individually, preferably in the morning (9 a.m.–1 p.m). To limit stress due to maternal separation, the time spent by the pup away from the mother and home cage was limited to the duration of each test. At the end of each test the pup was immediately put back in its home cage.

#### Ambulation Test

Ambulation was assessed every day between P6 and P12 to monitor the acquisition of walking proficiency. Each pup was placed on a flat, hard surface and the walking pattern was assessed over 1 min, according to previous scoring criteria (Feather-Schussler and Ferguson, 2016): 0 = no movement, 1 = crawling with asymmetric limb movement, 2 = slow crawling but symmetric limb movement, and 3 = fast crawling/walking.

#### Ear Development

The day of the opening of the ear canal, defined as a fully detached outer ear membrane, was recorded in pups aged P3–P5. A fixed score of 0, 1 or 2, according to the number of ears everted per animal was assigned.

#### Eyelid Opening

The day of the eyelid opening, defined as any visible break in the membrane covering the eye, was recorded in pups aged P12–P16. A fixed score of 0, 1 or 2, according to the number of eyelids opened per animal was assigned.

#### Righting Reflex

Every 2 days between P2 and P6, each pup was placed on its back on a flat, hard surface and kept immobile for 5 s. The pup was then released and the time taken to return to the upright position was recorded. Animals unable to perform the righting after 1 min were assigned a score of 60 s.

#### Acoustic Startle Reflex

Pups aged P10–P14 were examined daily to determine the day of reflex acquisition. A cell counter was used to generate an auditive stimulus and the presence (score: 1) or absence (score: 0) of a startle response was recorded.

#### Ultrasonic Vocalisations Communication

At P6, individual pups were placed on a cotton-padded dish into a thermo-controlled (26°C) soundproof chamber. USV were recorded for 5 min using the UltraSoundGate Condenser Microphone and 116 USB Audio device (Avisoft Bioacoustics), as described (Scattoni et al., 2009; Ferhat et al., 2016). Sonograms were analyzed with the Avisoft-SASLab Pro software (version 5.2.12, Avisoft Bioacoustics, Glienicke, Germany) using a 25 kHz cut-off frequency and a 5–10 ms element separation, as described in Ferhat et al. (2016). Automatedly identified USV identified using an automated process embedded in Avisoft-SASLab Pro software were then manually curated and the number and duration of each USV was extracted. We verified that there was no significant change in body temperature before and after USV recording using a thermosensor placed at the nape of the neck.

#### Olfactory Orientation

Each pup was separated from its litter and placed into the center of a rectangular-shaped transparent plastic box divided into three zones: the “maternal” zone on one side, the central zone and the “clean” zone on the opposite side from the maternal zone. The maternal zone contained freshly sampled maternal bedding, while the clean zone contained clean bedding. A Plexiglas odor separator was placed above the central zone to prevent the maternal smell from permeating. The movement of the pup was video-recorded over a period of 10 min. The ANY-maze video tracking software (reference) was used to determine: the latency to the first entry in the maternal bedding zone and the time spent in each of the three zones.

#### Exploratory Behavior

At P13, each pup was individually placed into a rectangular-shaped transparent plastic box. The movement of the pup was recorded over a period of 10 min and the videos were analyzed using the ANY-maze videotracking software to determine the total distance traveled and the time spent mobile. The exploratory index was then computed by dividing the distance by the time mobile.

### Collection of Samples

**FIGURE 1:**
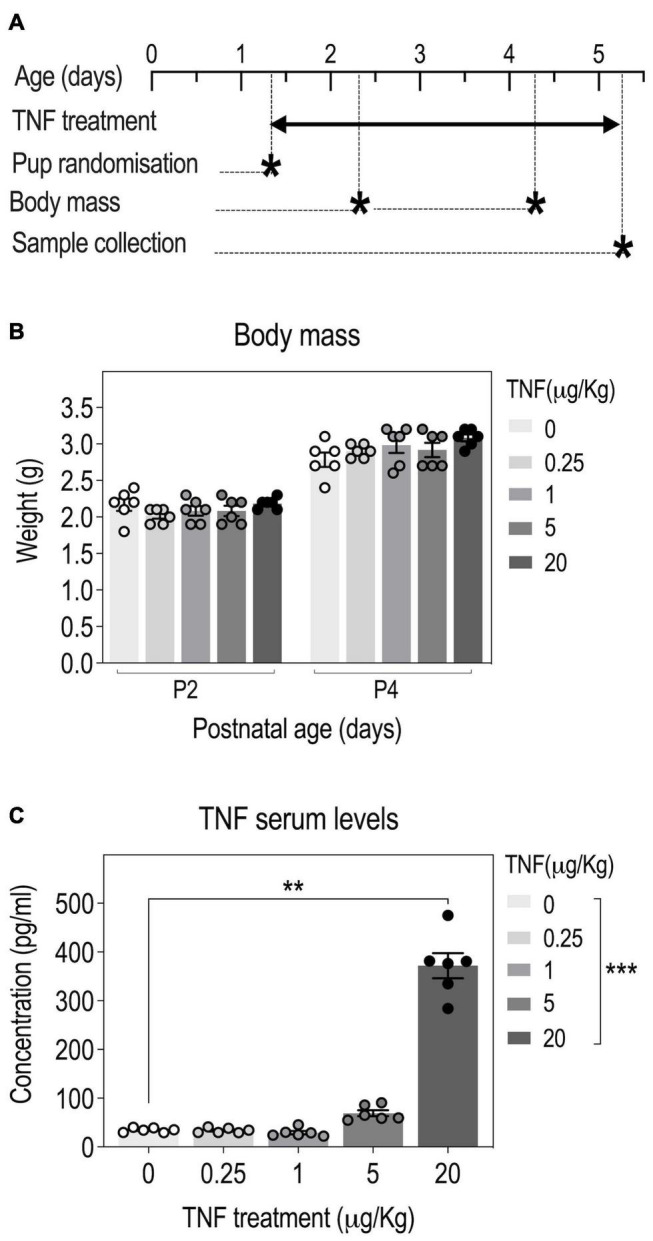
Tumor necrosis factor (TNF) perinatal injections in pups yield a dose-dependent increase in serum TNF concentrations. Timeline: OF-1 newborn pups were injected intraperitoneally with four different doses of TNF (0.25, 1, 5, and 20 μg/kg) or PBS (Vehicle, Veh) from P1 to P5 and serum TNF levels were measured at P5 **(A)**. Body weight of pups at P2 and P4; *n* = 6 Veh, *n* = 6 TNF; 2-way ANOVA: *p*(Treatment) = 0.4368, *p*(Postnatal age) < 0.0001, *p*(Interaction) = 0.0002 **(B)**. TNF serum levels at P5; data are presented as means ± SEM; *n* = 6 Veh, *n* = 6 TNF; Kruskal-Wallis test: *p*(Treatment) = 0.0001; Dunn’s *post*–*hoc* test for treatment effect: *p < 0.05, **p < 0.01, ***p < 0.001 **(C)**. Only statistically significant differences are presented.

**FIGURE 2:**
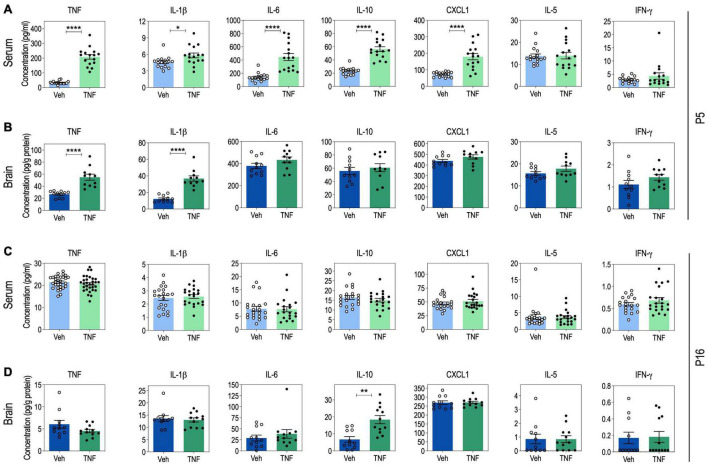
Serum and brain cytokine levels in tumor necrosis factor (TNF)-injected pups at P5 and P16. Serum cytokine levels at P5: *n* = 16 Vehicle (Veh), *n* = 16 TNF (20 mg/Kg) **(A)**. Brain cytokine levels at P5: *n* = 11 Veh, *n* = 11 TNF **(B)**. Serum cytokine levels at P16: for TNF, *n* = 30 Veh, *n* = 31 TNF. For IL-1β, IL-6, IL-10, IFN-γ, IL-5, and CXCL1, *n* = 20 Veh, *n* = 21 TNF **(C)**. Brain cytokine levels at P16: *n* = 11 Veh, *n* = 12 TNF (20 mg/Kg) **(D)**. For panels **(A–D)**, data are presented as means ± SEM. Mann-Whitney’s *U*-tests: *p < 0.05, **p < 0.01, and ****p < 0.0001. Only statistically significant differences are presented.

**FIGURE 3:**
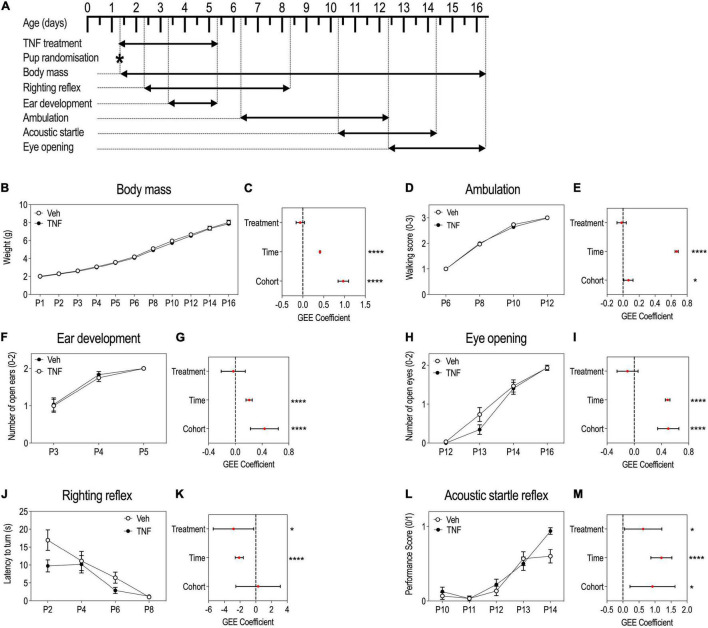
Tumor necrosis factor (TNF) impacts reflex acquisitions in pups but not overall growth and developmental milestones. Timeline of the experiment **(A)**. P1 to P16 body mass (BM) of pups injected with TNF (20 mg/Kg) or vehicle, presented as means ± SEM, note overlapping curves and reduced SEM **(B)**. GEE estimates, 95% CI and *p*-values associated with the effects of treatment, time and cohort on BM: *p*(Treatment) = 0.2183, *p*(Time) < 0.0001, *p*(Cohort) < 0.0001 **(C)**. Ambulation abilities scored from 0 to 3 in pups aged P6–P12, presented as means ± SEM, note overlapping curves and reduced SEM **(D)**. GEE estimates, 95% CI and *p*-values associated with the effects of treatment, time and cohort on ambulation abilities: *p*(Treatment) = 0.5604, *p*(Time) < 0.0001, and *p*(Cohort) = 0.0216 **(E)**. Ear development of pups aged P3–P5: a score of 0, 1 or 2 was set according to the number of ears everted/animal, presented as means ± SEM **(F)**. GEE estimates, 95% CI and *p*-values associated with the effects of treatment, time and cohort on ear development: *p*(Treatment) = 0.729, *p*(Time) < 0.0001, *p*(Cohort) < 0.0001 **(G)**. Eye opening of pups aged P12–P16: score of 0, 1 or 2 was set according to the number of eyes opened/animal, presented as means ± SEM **(H)**. GEE estimates, 95% CI and *p*-values associated with the effects of treatment, time and cohort on eye opening: *p*(Treatment) = 0.212, *p*(Time) < 0.0001, *p*(Cohort) < 0.0001 **(I)**. Righting reflex latency to turn of pups aged P2–P8, presented as means ± SEM **(J)**. GEE estimates, 95% CI and *p*-values associated with the effects of treatment, time and cohort on righting reflex acquisition: *p*(Treatment) = 0.0308, *p*(Time) < 0.0001, *p*(Cohort) = 0.840 **(K)**. Acoustic startle reflex of pups aged P10–P14: a score of either 0 or 1, where 1 is given to pups with startle and 0, to pups without startle, presented as means ± SEM **(L)**. GEE estimates, 95% CI and *p*-values associated with the effects of treatment, time and cohort on the acoustic startle acquisition: *p*(Treatment) = 0.0332, *p*(Time) < 0.0001, *p*(Cohort) = 0.0101 **(M)**. *n* = 29–30 PBS, *n* = 29–32 TNF depending on the test. *p < 0.05, ****p < 0.0001. Only statistically significant differences are presented.

**FIGURE 4:**
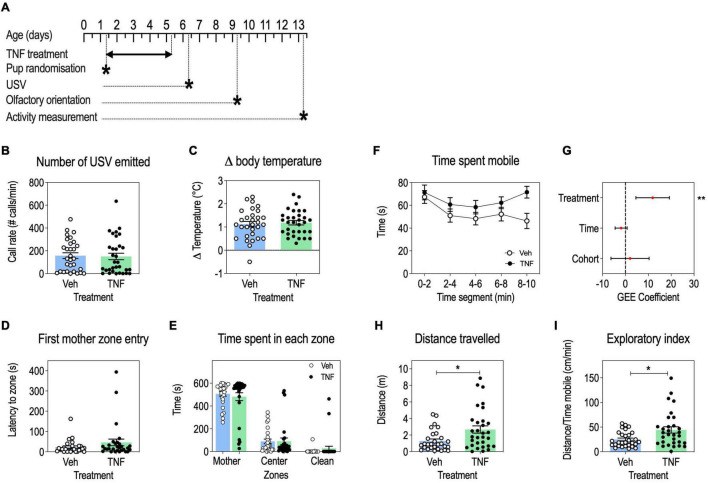
Tumor necrosis factor (TNF) increases locomotor activity and exploratory behavior in pups, but has no impact on USV communication, body temperature regulation and olfactory orientation. Timeline **(A)**. USV call rates at P6 in pups injected with TNF (20 mg/Kg) or vehicle, presented as means ± SEM, Mann-Whitney’s *U*-test: *p* > 0.05 **(B)**. Change in pup body temperature during USV recording, presented as means ± SEM, Mann-Whitney’s *U*-test: *p* > 0.05 **(C)**. Olfactory orientation at P9: latency to the first entry in the maternal bedding zone, presented as means ± SEM, Mann-Whitney’s *U*-test: *p* > 0.05 **(D)**; time spent in maternal bedding zone, center zone and clean bedding zone, presented as means ± SEM, 2-way ANOVA: *p*(Treatment) = 0.9602, *p*(Time) < 0.0001, *p*(Interaction) = 0.7046 **(E)**. Exploratory behavior at P13: time spent mobile in pups aged P13, by 2-min time segments, presented as means ± SEM **(F)**. GEE estimates, 95% CI and *p*-values associated with the effects of treatment, time and cohort on time spent mobile: *p*(Treatment) = 0.0014, *p*(Time) = 0.1632, *p*(Cohort) = 0.6345 **(G)**. Total distance traveled, presented as means ± SEM, Mann-Whitney’s *U*-test: *p*(Treatment) = 0.0157 **(H)**. Exploratory index, presented as means ± SEM, Mann-Whitney’s *U*-test: *p*(Treatment) = 0.0266 **(I)**. *n* = 29–30 Veh, *n* = 29–32 TNF depending on the test; *p < 0.05, **p < 0.01. Only statistically significant differences are presented.

Pups were sacrificed by decapitation at age P5 in the pilot cohort used to determine the optimal TNF dose (Figure 1) and one other independent cohort injected with TNF (20 μg/Kg) or vehicle used to determine cytokine levels in serum and brain at P5 (Figure 2A). The three cohorts injected with TNF (20 μg/Kg) or vehicle, that were subjected to behavioral testing (Figures 2B, 3, 4), were sacrificed at P16 by a lethal injection of anaesthetic. The blood was collected either from the trunk at the moment of decapitation (at P5), or by cardiac puncture (at P16) into clean 1.5 ml Eppendorf tubes and allowed to clot for 1 h at room temperature (RT). Blood was then centrifuged (10,000 × *g*, 4°C, 20 min) and serum was transferred to clean 0.5 ml Eppendorf tubes, immediately frozen in liquid nitrogen and stored at −80°C until further analysis. Whole brains were removed from the skull, parted in hemibrains, immediately snapped-frozen in liquid nitrogen, and stored at −80°C until further analysis.

### Serum and Brain Cytokine Measurements

Hemibrain samples were homogenized using a Dounce potter in a lysis buffer (50 mM Tris pH 7.4, NaCl 150 mM, MgCl_2_ 1.25 mM, Triton 0.5%) containing protease and phosphatase inhibitors (Pierce, ThermoFisher Scientific, Illkirch, France) and centrifuged (17,000 × *g*, 10 min, 4°C). The protein content of the resulting supernatant was dosed using the Bradford method (Biorad, Marnes-la-Coquette, France). Cytokines were measured in serum samples diluted to 1:4 and in 200 μg of brain proteins using either the V-PLEX^®^ Mouse TNF Kit for TNF single measures or the V-PLEX^®^ Pro-inflammatory Panel 1 Mouse Kit for multiplex measures (IFN-γ, IL-1β, IL-2, IL-4, IL-5, IL-6, IL-10, IL-12p70, CXCL1, and TNF), according to the manufacturer’s instructions (Meso Scale Diagnostics, Rockville, MD, United States). Briefly, eight serial dilutions of standards and buffer only (in duplicates) were run together with samples run in singlicate using the Sector Imager 2,400 plate reader (Meso Scale Diagnostics, Rockville, MD, United States). Concentrations of cytokines in each sample were interpolated from standard curves generated with a five-parameter logistic regression equation in Discovery Workbench 3.0 software (Meso Scale Diagnostics, Rockville, MD, United States). IL-2, IL-4, and IL-12p70 were below the Lower Limit of Detection (LLOD) in the majority of the samples and were therefore not considered.

### Statistics

#### Univariate Analysis

Data normality was assessed using the Kolmogorov-Smirnov test. For normal data, the two-tailed unpaired Student’s *t*-test was used. If normality was not reached after log-transformation, the non-parametric Mann-Whitney’s *U*-test was used. For multiple group comparisons, a two-way analysis of variance (ANOVA) with *post-hoc* Sidak’s correction for multiple comparisons was used. Statistical significance was set at *p*-value (*p*) lower than 0.05. All univariate analysis was performed using GraphPad Prism version 6.00 for Windows (Graphpad Software, San Diego, CA, United States). Details regarding statistical analyses outcomes are reported in Supplementary Table 1.

#### Multivariable Analysis of Developmental and Behavioral Outcomes

To account for correlations between repeated measures on the same individual, and random effects due to the cohort and developmental time of measurement, we used the generalized estimating equations (GEE) approach to model each outcome, considering treatment, cohort and time as covariates (Burton et al., 1998; Aktas Samur et al., 2014). GEE can be considered as an extension of generalized linear models (GLM) applied to longitudinal data (Burton et al., 1998). Instead of modeling the within-individual covariance structure, GEE models the average response, thereby estimating the changes in average response for every one-unit increase in a covariate across the population. Of note, the GEE allows to capture global effects of the variables (treatment, time, cohort) on the outcomes of interest (developmental milestones, reflex acquisition or behavior), and does not allow to determine effects at defined time points. The R Package geepack was used to implement GEE (Højsgaard et al., 2006). GEE outcomes are reported in Supplementary Table 1.

## Results

### Perinatal Tumor Necrosis Factor Injections Yield Increased Serum and Brain Levels of Tumor Necrosis Factor at P5

To determine the optimal dose of TNF to use in the study, we performed a pilot experiment in which mouse pups were injected with TNF doses ranging from 0.25 to 20 μg/Kg (Figure 1A). Even when injected with the highest TNF dose, all pups survived and there was no overt visual sign of inflammation (redness, swelling) on the injected flank. There was no significant effect of TNF treatment on body weight in all groups, suggesting that TNF treatment did not impair growth (Figure 1B). Compared to saline-injected mice, pups injected with low doses of TNF (0.25, 1 μg/Kg) did not exhibit changes in circulating levels of TNF at P5. For pups injected with higher doses, 5 and 20 μg/Kg, there was a dose-dependent increase in serum TNF levels at P5, respectively (Figure 1C). Based on these results, the highest dose of TNF (20 μg/Kg) was used in further experiments.

### Perinatal Tumor Necrosis Factor Injections in Pups Induce Changes in Cytokine Levels in Serum and Brain

At P5, pups injected with TNF (20 μg/Kg) exhibited increased serum levels of TNF, IL-1β, IL-6, IL-10, and CXCL1, as compared to vehicle-injected pups, while IL-5 and IFN-g levels were not impacted (Figure 2A). This was accompanied by an increase of TNF and IL-1β levels in the brain of TNF-injected pups, compared to vehicle-injected pups, while IL-6, IL-10, CXCL1, IL-5, and IFN-g levels were not impacted (Figure 2B).

At P16, vehicle- and TNF-injected pups exhibited similar levels of TNF, IL-1β, IL-6, CXCL1, IL-5, and IFN-γ in both serum and brain samples (Figures 2C,D). In contrast, there was a twofold increase in the brain levels of IL-10 in TNF-injected pups, as compared to vehicle-injected pups, while serum IL-10 was not impacted (Figures 2C,D).

These results suggest that perinatal injections of TNF from P1 to P5 induce an immediate acute systemic inflammation, that spreads to the brain. However, by P16, inflammation has resumed and only the anti-inflammatory cytokine IL-10 appears overexpressed.

### Tumor Necrosis Factor-Injected Pups Exhibit a Precocious Acquisition of Sensorimotor Reflexes

To monitor the behavioral consequences of perinatal systemic inflammation induced by TNF treatment, pups injected with TNF (20 μg/Kg) or vehicle from P1 to P5 were subjected to a battery of tests (Figure 3A). There were significant effects of time and cohort on BM, but no significant effect of TNF treatment (Figures 3B,C), suggesting that TNF did not impact general growth. Furthermore, TNF treatment did not impact the acquisition of developmental milestones: ambulation abilities progression (Figure 3D,E), or kinetics for ear eversion (Figures 3F,G) or eyelid opening (Figures 3H,I), while there were significant effects of time and cohort variables. This suggested that TNF did not induce gross developmental changes.

However, TNF treatment had a significant effect on the acquisition of early reflexes over time (Figures 3J–M). Both the righting reflex (Figures 3J,K) and the acoustic startle reflex (Figures 3L,M) occurred at earlier stages in TNF-injected pups compared to control animals. At P2, TNF-injected pups took almost half the time to resume a normal body position than control pups (Figure 3J). By P14, all the TNF-injected mice had acquired the acoustic startle reflex, while only 50% of the control pups had acquired it (Figure 3L).

### Tumor Necrosis Factor-Injected Pups Exhibit Increased Locomotor Activity and Exploratory Behavior

We further studied the impact of perinatal TNF treatment (Figure 4A) on communication abilities by measuring USV emissions upon a short maternal separation. TNF-treated pups emitted a similar number of vocalizations than vehicle-injected mice (Figure 4B). Moreover, there was no difference in the loss of body temperature between TNF treatment and control during the USV test (Figure 4C) suggesting that TNF did not induce thermoregulation issues which could interfere with USV measures.

In the olfactory orientation test, TNF- and vehicle-injected pups performed equally well as measured by the latency to first reach maternal bedding (Figure 4D) and the equal time spent in the three zones of the set-up (Figure 4E). This indicated that sensorimotor processing was similar in both groups for this task.

Regarding locomotor activity, TNF-injected pups spent more time mobile (Figures 4F,G) and traveled longer distances (Figure 4H) than vehicle-injected pups, but there was no effect of time or cohort variables on these parameters. This suggests that TNF increased locomotor activity in pups. Given that by P12 both TNF-injected and control pups had acquired a mature walking pattern (Figures 3D,E), it may reflect an increased exploratory activity in TNF-injected pups, as shown by an increased exploratory index as compared to control pups (Figure 4I).

## Discussion

### Tumor Necrosis Factor Perinatal Injections in the Periphery Trigger Transient Changes in Peripheral and Brain Cytokine Levels

Our study is based on gain-of-function experiments in which mouse pups are injected intraperitoneally with recombinant TNF daily from P1 to P5. We show that this yielded a robust increase in serum TNF levels at P5. Concomitantly, we observed an increase in the serum levels of the pro-inflammatory cytokines IL-1β and IL-6, the chemokine CXCL1, and the anti-inflammatory cytokine IL-10. This suggests that exogenous TNF injected peripherally triggers the secretion of other peripheral cytokines.

The effects of TNF injections appear more selective in the brain. Indeed, we show that, at P5, TNF-injected pups exhibit increased brain levels of both TNF and IL- 1β, while other cytokines are unimpacted. Since it was demonstrated in 3-days old rat pups that radiolabeled soluble TNF injected peripherally crossed the BBB using a saturable transport system (Gutierrez et al., 1993), it is likely that at least a fraction of the recombinant TNF that we injected peripherally crossed the BBB, contributing to the twofold increase in TNF brain levels. Even though peripheral IL-1β is also able to cross the BBB (Banks et al., 1991), it is more likely that the increase in brain IL-1β levels is due to a local brain production, possibly *via* microglia, as the magnitude of IL-1β increase in the brain (threefold) is higher than in serum (1.27-fold). Upon TNF injections, the lack of elevation of other cytokines in the brain at P5 (including pro-inflammatory cytokines) suggests that their transfer from the periphery to the brain or their local production in the brain relies on distinct regulatory processes, independently of TNF or IL-1β.

The increases in pro-inflammatory cytokine levels are transient, as, by P16, the levels of all these cytokines have returned to control levels in brain or serum. By P16, the only cytokine exhibiting higher levels is the anti-inflammatory cytokine IL-10. It is possible that IL-10 elevation is consecutive to the transient increase in pro-inflammatory cytokines TNF and IL-1β in the brain at P5. Indeed, IL-10 is a potent anti-inflammatory cytokine secreted to resume inflammation notably by inhibiting TNF and IL-1β expression by activated immune cells and microglia (Saraiva et al., 2020).

Of note, classically activated M1-polarized microglia secretes high levels of the pro-inflammatory cytokines TNF and IL-1β in response to brain insults (Amici et al., 2017). In contrast, M2-polarized microglia promotes tissue repair *via* release of anti-inflammatory cytokines such as IL-10 and low levels of pro-inflammatory cytokines (Amici et al., 2017). We can envision that at P5, repeated TNF injections have skewed microglia toward the M1 state and that by P16, when TNF and IL-1β are no longer elevated, the microglia has transitioned to the M2 phenotype and secretes IL-10. This could explain why IL-10 is the only cytokine remaining elevated in the brain of TNF-injected animals at this stage.

Tumor necrosis factor brain levels are constitutively low and TNF homeostatic function should be studied in conditions of modest increase (3–5-fold) (Heir and Stellwagen, 2020), in contrast to the massive increase in peripheral cytokines levels observed upon systemic inflammation resulting from immune activation obtained for example after LPS or poly(I:C) challenge. We therefore consider that our model is suitable to study the impact of transient systemic and central increases in TNF during early postnatal neurodevelopment, as recently pointed (Heir and Stellwagen, 2020).

Previous studies have shown that sTNF triggers TNFR1 signaling transduction pathway and that all cells of the brain parenchyma, including neurons, astrocytes, oligodendrocytes and microglia, as well as epithelial cells of the BBB express TNFR1 (Probert, 2015; Holbrook et al., 2019). Therefore, the effects of TNF injection on the acquisition of sensorimotor reflexes and exploratory behavior may be explained by a direct effect of TNF on several cell types *via* TNFR1. Also, IL-1β is increased in the brain at P5 in TNF-injected pups. Since IL-1β receptor IL1R1 is expressed in endothelial cells, neurons and at very low levels in astrocytes (Liu et al., 2019), it is possible that the effects on TNF injection are partly mediated by increased signaling *via* IL-1b.

### Tumor Necrosis Factor Impacts Reflex Acquisition Trajectories, Locomotor Activity, and Exploratory Behavior in Mouse Pups

Tumor necrosis factor-injected pups exhibit a precocious acquisition of reflexes as well as increased locomotor activity and exploratory behavior. General growth as well as other behaviors such as USV communication or olfactory orientation were not impacted, suggesting a selective impact of TNF during neurodevelopment. Previous studies have shown that TNF mediates homeostatic synaptic plasticity (Heir and Stellwagen, 2020). The impact of TNF we observe on sensorimotor reflexes and exploratory behavior could be mediated by this form of plasticity, acknowledged as critical for the developmental plasticity of sensory systems (Heir and Stellwagen, 2020).

The righting reflex rectifies the orientation of the pup when it is placed supine on its back. In early developmental stages, the reflex essentially relies on somatosensory inputs (tactile stimuli on the body and head), and does not involve visual inputs since eyelids are not opened or vestibular inputs, as the vestibular system is not mature yet at P2 (Jamon, 2014). The cerebellum is a key region for integrating somatosensory inputs and correct posture *via* motor neurons of the spinal cord which control muscular movements to right the body (Manzoni, 2007). It may be envisioned that TNF-injected pups may have undergone an earlier development of the cerebellar or spinal neurons coordinating the righting reflex at early stages. TNF was shown to enhance the intrinsic excitability of cerebellar Purkinje cells in the juvenile rat cerebellum (Kuno et al., 2018). This could contribute to earlier acquisition of the righting reflex by a better integration of proprio-tactile cues and a quicker coordination of movement. Later in development it could increase locomotor activity and exploratory behavior, as we have shown in TNF-injected pups.

The startle response is an unconscious defensive response to a sudden stimulus, in our case acoustic. In mouse pups, the startle reflex yields a sudden extension of the head and fore and hind limbs which are then withdrawn to reach a crouched position. The neural pathway involved is well described and involves first a synapse from the auditory nerve fibers in the ear to the cochlear root neurons (CRN), the first acoustic neurons of the central nervous system (Davis, 1984). The CRN axons then synapse on neurons in the *nucleus reticularis pontis caudalis* (PnC), located in the pons of the brainstem. Finally, the PnC axons synapse to spinal motor neurons to induce the startle (Davis, 1984). It is possible that TNF-injected pups have developed earlier axonal projections of CRNs or PnC neurons, causing the reflex to occur earlier.

At P13, pups have hardly opened their eyelids and spatial information processing is still immature (Ricceri et al., 2000). At this stage, pups mostly rely on sensory inputs from palpation with their whiskers for guidance of exploratory motor behavior (Arakawa and Erzurumlu, 2015). In the juvenile male rat, TNF increases the intrinsic excitability of cerebellar Purkinje cells (Shim et al., 2018). Furthermore, a recent study has demonstrated that whisker reflex adaptation was facilitated by potentiation of cerebellar Purkinje cells (Romano et al., 2018). It is possible that perinatal injection of TNF could modify the course of development of Purkinje cells, contributing to increase palpation whisking-driven exploratory behavior at this stage.

Whether the changes we observed reveal a beneficial or negative neurodevelopmental impact of TNF remains to be determined. On one hand, a precocious acquisition of reflexes and increase in exploratory behavior may reflect developmental precocity and could confer selective advantages to TNF-injected pups. On the other hand, faster reflex acquisition can reflect an inappropriate neurodevelopmental timing which translate into hyperactivity in TNF-injected pups. On the whole, our data support the notion that the pro-inflammatory cytokine TNF selectively impacts neurodevelopmental trajectories.

### Comparison With Other Models of Perinatal Inflammation

Several studies have monitored the impact of perinatal inflammation on neurodevelopment and behavior, however, most of them have focused on behavioral outcomes in adulthood. One previous study reported that perinatal treatment with the pro-inflammatory cytokine IL-1β, twice daily from P1 to P5 (at 10 μg/Kg), increased circulating levels of IL-1β and TNF-α (Favrais et al., 2011). This was accompanied by neuroinflammation, white matter lesions, and impaired oligodendrocytes maturation (Favrais et al., 2011). Adult mice injected perinatally with IL-1β also exhibited cognitive deficits in the novel object recognition task, but no defects in exploratory behavior by P30 (Favrais et al., 2011). However, the impact of IL-1β injection on behavior was not studied during the first two postnatal weeks of life, impeding further comparison with our results obtained after TNF injection. Another study showed that repeated perinatal injections of IL-1β, IL-6, IL-9, and TNF (twice a day at 20 μg/Kg) sensitized the brain to further excitotoxic brain lesions at P5 (Dommergues et al., 2003). However, the impact on early behavior was not assessed. Finally, early postnatal behavioral changes were reported in the maternal immune activation (MIA) model. Pups exposed *in utero* to MIA exhibit USV communication deficits and hypoactivity, and elevated TNF levels in serum at P16 (Paraschivescu et al., 2020). Hence, there is no overlap with the behavioral changes we observe in TNF-injected pups, hindering further comparison of our data with this inflammatory model.

### Methodological and Statistical Choices for Behavioral Data Collection and Analyses

Genetic, environmental factors, maternal effect and litter effects are acknowledged confounding variables in developmental and behavioral studies. We accounted for environmental and maternal effects by adapting our experimental procedures, notably by pooling pups of different litters to form new litters and including TNF- and vehicle-treated pups in the same litters. Our experimental model is the OF1 outbred stock obtained from “a closed population of genetically variable animals that is bred to maintain maximum heterozygosity” as defined in Chia et al. (2005). Although outbred stocks present the advantage to mimic more closely human populations, outbred stocks bear recessive mutations that may affect experimental results and lower treatment effect size. This genetic variation may affect behavioral responses, since a stock may contain a mixture of homozygous, heterozygous and wildtype pups (Chia et al., 2005). The fact that we randomized pups from P1 to form new litters minimizes the possible confounding effect of genetics in our model.

To account for the effect of variables (e.g., developmental time, cohort) which we could not experimentally control, but which, besides TNF treatment, could impact developmental and behavioral outcomes, we used the Generalized Estimating Equations (GEE) model. This modeling strategy is particularly adapted to the study of outcomes measured repeatedly over time on the same individuals and subjected to confounding effects. In place of more classical regression methods which estimate the effect of changing one or more covariates on a given individual, GEE focuses on estimating “population-averaged” effects (Burton et al., 1998; Aktas Samur et al., 2014). GEE can be considered as an extension of generalized linear models (GLM) applied to longitudinal data (Burton et al., 1998; Aktas Samur et al., 2014). Also, compared to mixed-effects models, GEE appears generally better suited to analyse outcomes measured over time in animal studies (Hubbard et al., 2010). Hence, this approach is robust to model misspecifications and small sample size, which is generally the norm in animal studies (Hubbard et al., 2010). Our study underlines the potential of GEE approaches to model the effect of variables on developmental and behavioral outcomes in animal studies.

### Limitations of the Study

First, our study is restricted to males. A large body of evidence suggests that males are more at risk than females for neurodevelopmental disorders (Davis and Pfaff, 2014). Likewise, several pre-clinical studies in rodents have shown a sexual dimorphism in neurodevelopmental mechanisms and in sensitivity to developmental brain insults (DiPietro and Voegtline, 2017). Therefore, it remains to be determined whether TNF has similar impact in female pups. Second, pups were separated daily from the mother for testing. This stress could have contributed to exacerbating the effects of the TNF treatment. Of note, vehicle-injected animals were manipulated identically to TNF-injected animals and therefore subjected to the same stress. Third, future work should address whether TNF perinatal injection induced long-lasting behavioral changes beyond P14, and notably in adult animals. Fourth, our study relies on pups from OF1 outbred stock. The possibility to generalize our data to inbred mouse strains such as C57Bl/6J should be explored in follow-up studies.

## Conclusion

Our study revealed the selective early life impact of perinatal TNF on reflex acquisition, as well as locomotor activity. This suggests that TNF can modulate early developmental trajectories and complement previous behavioral studies performed in adult animals. Future work is required to understand the astrocytic, microglial or neuronal pathways underlying the effects of TNF on early development, and whether they converge on neurodevelopmental pathways generally impacted by ELS.

## Data Availability Statement

The raw data supporting the conclusions of this article will be made available by the authors, without undue reservation.

## Ethics Statement

The animal study was reviewed and approved by the Ministère de l’Enseignement Supérieur et de la Recherche.

## Author Contributions

LD and NG designed the project. CP carried out the experiments. CP, SB, and LD analyzed and interpreted the data. JV and PG advised on experimental design and contributed methods. LD and CP wrote the manuscript. CP, JV, SB, and NG revised the manuscript. All authors contributed to the article and approved the submitted version.

## Conflict of Interest

The authors declare that the research was conducted in the absence of any commercial or financial relationships that could be construed as a potential conflict of interest.

## Publisher’s Note

All claims expressed in this article are solely those of the authors and do not necessarily represent those of their affiliated organizations, or those of the publisher, the editors and the reviewers. Any product that may be evaluated in this article, or claim that may be made by its manufacturer, is not guaranteed or endorsed by the publisher.

## References

[B1] Aktas Samur A. Coskunfirat N. Saka O. (2014). Comparison of predictor approaches for longitudinal binary outcomes: application to anesthesiology data. *PeerJ* 2:e648. 10.7717/peerj.648 25374787 PMC4217193

[B2] Amici S. A. Dong J. Guerau-de-Arellano M. (2017). Molecular mechanisms modulating the phenotype of macrophages and microglia. *Front. Immunol.* 8:1520. 10.3389/fimmu.2017.01520 29176977 PMC5686097

[B3] Arakawa H. Erzurumlu R. S. (2015). Role of whiskers in sensorimotor development of C57BL/6 mice. *Behav. Brain Res.* 287 146–155. 10.1016/j.bbr.2015.03.040 25823761 PMC4430837

[B4] Arnett H. A. Mason J. Marino M. Suzuki K. Matsushima G. K. Ting J. P. (2001). TNF alpha promotes proliferation of oligodendrocyte progenitors and remyelination. *Nat. Neurosci.* 4 1116–1122. 10.1038/nn738 11600888

[B5] Banks W. A. Ortiz L. Plotkin S. R. Kastin A. J. (1991). Human interleukin (IL) 1 alpha, murine IL-1 alpha and murine IL-1 beta are transported from blood to brain in the mouse by a shared saturable mechanism. *J. Pharmacol. Exp. Ther.* 259 988–996. 1762091

[B6] Beattie E. C. Stellwagen D. Morishita W. Bresnahan J. C. Ha B. K. Von Zastrow M. (2002). Control of synaptic strength by glial TNFalpha. *Science* 295 2282–2285. 10.1126/science.1067859 11910117

[B7] Bernardino L. Agasse F. Silva B. Ferreira R. Grade S. Malva J. O. (2008). Tumor necrosis factor-alpha modulates survival, proliferation, and neuronal differentiation in neonatal subventricular zone cell cultures. *Stem Cells* 26 2361–2371. 10.1634/stemcells.2007-0914 18583543

[B8] Biesmans S. Bouwknecht J. A. Ver Donck L. Langlois X. Acton P. D. De Haes P. (2015). Peripheral administration of tumor necrosis factor-alpha induces neuroinflammation and sickness but not depressive-like behavior in mice. *Biomed Res. Int.* 2015:716920. 10.1155/2015/716920 26290874 PMC4531164

[B9] Burton P. Gurrin L. Sly P. (1998). Extending the simple linear regression model to account for correlated responses: an introduction to generalized estimating equations and multi-level mixed modelling. *Stat. Med.* 17 1261–1291. 10.1002/(sici)1097-0258(19980615)17:11<1261::aid-sim846>3.0.co;2-z 9670414

[B10] Camara M. L. Corrigan F. Jaehne E. J. Jawahar M. C. Anscomb H. Baune B. T. (2015). Effects of centrally administered etanercept on behavior, microglia, and astrocytes in mice following a peripheral immune challenge. *Neuropsychopharmacology* 40 502–512. 10.1038/npp.2014.199 25103178 PMC4443965

[B11] Cattane N. Richetto J. Cattaneo A. (2020). Prenatal exposure to environmental insults and enhanced risk of developing schizophrenia and autism spectrum disorder: focus on biological pathways and epigenetic mechanisms. *Neurosci. Biobehav. Rev.* 117 253–278. 10.1016/j.neubiorev.2018.07.001 29981347

[B12] Chia R. Achilli F. Festing M. F. Fisher E. M. (2005). The origins and uses of mouse outbred stocks. *Nat. Genet.* 37 1181–1186. 10.1038/ng1665 16254564

[B13] Connor T. J. Song C. Leonard B. E. Merali Z. Anisman H. (1998). An assessment of the effects of central interleukin-1beta, -2, -6, and tumor necrosis factor-alpha administration on some behavioural, neurochemical, endocrine and immune parameters in the rat. *Neuroscience* 84 923–933. 10.1016/s0306-4522(97)00533-2 9579794

[B14] Dantzer R. O’Connor J. C. Freund G. G. Johnson R. W. Kelley K. W. (2008). From inflammation to sickness and depression: when the immune system subjugates the brain. *Nat. Rev. Neurosci.* 9 46–56. 10.1038/nrn2297 18073775 PMC2919277

[B15] Davis E. P. Pfaff D. (2014). Sexually dimorphic responses to early adversity: implications for affective problems and autism spectrum disorder. *Psychoneuroendocrinology* 49 11–25. 10.1016/j.psyneuen.2014.06.014 25038479 PMC4165713

[B16] Davis M. (1984). “Neural mechanisms of startle behavior,” in *The Mammalian Startle Response*, ed. Eaton R. C. (Boston, MA: Springer), 287–351. 10.1126/science.229.4709.158

[B17] DiPietro J. A. Voegtline K. M. (2017). The gestational foundation of sex differences in development and vulnerability. *Neuroscience* 342 4–20. 10.1016/j.neuroscience.2015.07.068 26232714 PMC4732938

[B18] Dommergues M. A. Plaisant F. Verney C. Gressens P. (2003). Early microglial activation following neonatal excitotoxic brain damage in mice: a potential target for neuroprotection. *Neuroscience* 121 619–628. 10.1016/s0306-4522(03)00558-x 14568022

[B19] Favrais G. van de Looij Y. Fleiss B. Ramanantsoa N. Bonnin P. Stoltenburg-Didinger G. (2011). Systemic inflammation disrupts the developmental program of white matter. *Ann. Neurol.* 70 550–565. 10.1002/ana.22489 21796662

[B20] Feather-Schussler D. N. Ferguson T. S. (2016). A battery of motor tests in a neonatal mouse model of cerebral palsy. *J. Vis. Exp.* 117:53569. 10.3791/53569 27842358 PMC5226120

[B21] Ferhat A. T. Torquet N. Le Sourd A. M. de Chaumont F. Olivo-Marin J. C. Faure P. (2016). Recording mouse ultrasonic vocalizations to evaluate social communication. *J. Vis. Exp.* 112:53871. 10.3791/53871 27341321 PMC4927756

[B22] Fox W. M. (1965). Reflex-ontogeny and behavioural development of the mouse. *Anim. Behav.* 13 234–241. 10.1016/0003-3472(65)90041-2 5835840

[B23] Garay P. A. Hsiao E. Y. Patterson P. H. McAllister A. K. (2013). Maternal immune activation causes age- and region-specific changes in brain cytokines in offspring throughout development. *Brain Behav. Immun.* 31 54–68. 10.1016/j.bbi.2012.07.008 22841693 PMC3529133

[B24] Garre J. M. Silva H. M. Lafaille J. J. Yang G. (2017). CX3CR1^+^ monocytes modulate learning and learning-dependent dendritic spine remodeling via TNF-alpha. *Nat. Med.* 23 714–722. 10.1038/nm.4340 28504723 PMC5590232

[B25] Golan H. Levav T. Mendelsohn A. Huleihel M. (2004). Involvement of tumor necrosis factor alpha in hippocampal development and function. *Cereb. Cortex* 14 97–105. 10.1093/cercor/bhg108 14654461

[B26] Gough P. Myles I. A. (2020). Tumor necrosis factor receptors: pleiotropic signaling complexes and their differential effects. *Front. Immunol.* 11:585880. 10.3389/fimmu.2020.585880 33324405 PMC7723893

[B27] Gutierrez E. G. Banks W. A. Kastin A. J. (1993). Murine tumor necrosis factor alpha is transported from blood to brain in the mouse. *J. Neuroimmunol.* 47 169–176. 10.1016/0165-5728(93)90027-v 8370768

[B28] Hayley S. Wall P. Anisman H. (2002). Sensitization to the neuroendocrine, central monoamine and behavioural effects of murine tumor necrosis factor-alpha: peripheral and central mechanisms. *Eur. J. Neurosci.* 15 1061–1076. 10.1046/j.1460-9568.2002.01936.x 11918665

[B29] Heir R. Stellwagen D. (2020). TNF-mediated homeostatic synaptic plasticity: from *in vitro* to *in vivo* Models. *Front. Cell. Neurosci.* 14:565841. 10.3389/fncel.2020.565841 33192311 PMC7556297

[B30] Heyser C. J. (2004). Assessment of developmental milestones in rodents. *Curr. Protoc. Neurosci.* Chapter 8:Unit 8.18. 10.1002/0471142301.ns0818s25 18428605

[B31] Højsgaard S. Halekoh U. Yan J. (2006). The R package geepack for generalized estimating equations. *J. Stat. Softw.* 15 1–11.

[B32] Holbrook J. Lara-Reyna S. Jarosz-Griffiths H. McDermott M. (2019). Tumour necrosis factor signalling in health and disease. *F1000Res* 8:F1000 Faculty Rev-111. 10.12688/f1000research.17023.1 30755793 PMC6352924

[B33] Hubbard A. E. Ahern J. Fleischer N. L. Van der Laan M. Lippman S. A. Jewell N. (2010). To GEE or not to GEE: comparing population average and mixed models for estimating the associations between neighborhood risk factors and health. *Epidemiology* 21 467–474. 10.1097/EDE.0b013e3181caeb90 20220526

[B34] Jamon M. (2014). The development of vestibular system and related functions in mammals: impact of gravity. *Front. Integr. Neurosci.* 8:11. 10.3389/fnint.2014.00011 24570658 PMC3916785

[B35] Kaneko M. Stellwagen D. Malenka R. C. Stryker M. P. (2008). Tumor necrosis factor-alpha mediates one component of competitive, experience-dependent plasticity in developing visual cortex. *Neuron* 58 673–680. 10.1016/j.neuron.2008.04.023 18549780 PMC2884387

[B36] Kaster M. P. Gadotti V. M. Calixto J. B. Santos A. R. Rodrigues A. L. (2012). Depressive-like behavior induced by tumor necrosis factor-alpha in mice. *Neuropharmacology* 62 419–426. 21867719 10.1016/j.neuropharm.2011.08.018

[B37] Kriegler M. Perez C. DeFay K. Albert I. Lu S. D. (1988). A novel form of TNF/cachectin is a cell surface cytotoxic transmembrane protein: ramifications for the complex physiology of TNF. *Cell* 53 45–53. 10.1016/0092-8674(88)90486-2 3349526

[B38] Kuno T. Hirayama-Kurogi M. Ito S. Ohtsuki S. (2018). Reduction in hepatic secondary bile acids caused by short-term antibiotic-induced dysbiosis decreases mouse serum glucose and triglyceride levels. *Sci. Rep.* 8:1253. 10.1038/s41598-018-19545-1 29352187 PMC5775293

[B39] Lewitus G. M. Pribiag H. Duseja R. St-Hilaire M. Stellwagen D. (2014). An adaptive role of TNFalpha in the regulation of striatal synapses. *J. Neurosci.* 34 6146–6155. 10.1523/JNEUROSCI.3481-13.2014 24790185 PMC6608116

[B40] Liu B. Zupan B. Laird E. Klein S. Gleason G. Bozinoski M. (2014). Maternal hematopoietic TNF, via milk chemokines, programs hippocampal development and memory. *Nat. Neurosci.* 17 97–105. 10.1038/nn.3596 24292233 PMC6169993

[B41] Liu X. Nemeth D. P. McKim D. B. Zhu L. DiSabato D. J. Berdysz O. (2019). Cell-type-specific interleukin 1 receptor 1 signaling in the brain regulates distinct neuroimmune activities. *Immunity* 50 317–333.e6. 30683620 10.1016/j.immuni.2018.12.012 PMC6759085

[B42] Manzoni D. (2007). The cerebellum and sensorimotor coupling: looking at the problem from the perspective of vestibular reflexes. *Cerebellum* 6 24–37. 10.1080/14734220601132135 17366264

[B43] Paraschivescu C. Barbosa S. Lorivel T. Glaichenhaus N. Davidovic L. (2020). Cytokine changes associated with the maternal immune activation (MIA) model of autism: a penalized regression approach. *PLoS One* 15:e0231609. 10.1371/journal.pone.0231609 32760152 PMC7410235

[B44] Probert L. (2015). TNF and its receptors in the CNS: the essential, the desirable and the deleterious effects. *Neuroscience* 302 2–22. 10.1016/j.neuroscience.2015.06.038 26117714

[B45] Ranson A. Cheetham C. E. Fox K. Sengpiel F. (2012). Homeostatic plasticity mechanisms are required for juvenile, but not adult, ocular dominance plasticity. *Proc. Natl. Acad. Sci. U.S.A.* 109 1311–1316. 10.1073/pnas.1112204109 22232689 PMC3268335

[B46] Ricceri L. Colozza C. Calamandrei G. (2000). Ontogeny of spatial discrimination in mice: a longitudinal analysis in the modified open-field with objects. *Dev. Psychobiol.* 37 109–118. 10.1002/1098-2302(200009)37:2<109::aid-dev6>3.0.co;2-d 10954836

[B47] Romano V. De Propris L. Bosman L. W. Warnaar P. Ten Brinke M. M. Lindeman S. (2018). Potentiation of cerebellar Purkinje cells facilitates whisker reflex adaptation through increased simple spike activity. *Elife* 7:e38852. 10.7554/eLife.38852 30561331 PMC6326726

[B48] Santello M. Bezzi P. Volterra A. (2011). TNFalpha controls glutamatergic gliotransmission in the hippocampal dentate gyrus. *Neuron* 69 988–1001. 10.1016/j.neuron.2011.02.003 21382557

[B49] Saraiva M. Vieira P. O’Garra A. (2020). Biology and therapeutic potential of interleukin-10. *J. Exp. Med.* 217:e20190418. 10.1084/jem.20190418 31611251 PMC7037253

[B50] Scattoni M. L. Crawley J. Ricceri L. (2009). Ultrasonic vocalizations: a tool for behavioural phenotyping of mouse models of neurodevelopmental disorders. *Neurosci. Biobehav. Rev.* 33 508–515. 10.1016/j.neubiorev.2008.08.003 18771687 PMC2688771

[B51] Shim H. G. Jang S. S. Kim S. H. Hwang E. M. Min J. O. Kim H. Y. (2018). TNF-alpha increases the intrinsic excitability of cerebellar Purkinje cells through elevating glutamate release in Bergmann Glia. *Sci. Rep.* 8:11589. 10.1038/s41598-018-29786-9 30072733 PMC6072779

[B52] Stellwagen D. Malenka R. C. (2006). Synaptic scaling mediated by glial TNF-alpha. *Nature* 440 1054–1059. 10.1038/nature04671 16547515

[B53] Zupan B. Liu B. Taki F. Toth J. G. Toth M. (2017). Maternal brain TNF-alpha programs innate fear in the offspring. *Curr. Biol.* 27 3859–3863.e3. 10.1016/j.cub.2017.10.071 29199072 PMC6170164

